# Preoperative Lateralization and Diagnostic Value of Selective Bilateral Internal Jugular Venous Sampling in Primary Hyperparathyroidism: Single-Center Experience

**DOI:** 10.3390/medicina60030507

**Published:** 2024-03-19

**Authors:** Anastasija Solodjankina, Aina Kratovska, Sanita Ponomarjova, Patricija Ivanova, Reza Mohammadian

**Affiliations:** 1 Faculty of Medicine, Riga Stradinš University, LV-1007 Riga, Latvia; anastasija.solodjankina@gmail.com (A.S.); patricija.ivanova@lu.lv (P.I.); 2 Department of Interventional Radiology, Riga East University Hospital, LV-1038 Riga, Latvia; aina.kratovska@rsu.lv (A.K.); sanita.ponomarjova@rsu.lv (S.P.); 3 Department of Radiology, Riga Stradinš University, LV-1007 Riga, Latvia; 4 Department of Vascular Surgery, Riga East University Hospital, University of Latvia, LV-1586 Riga, Latvia

**Keywords:** primary hyperparathyroidism, selective venous sampling (SVS), parathyroid adenoma, non-invasive imaging

## Abstract

*Background and Objectives*: Primary hyperparathyroidism (pHPT) is a common endocrine disorder caused by excessive production of parathyroid hormone (PTH) leading to elevated calcium levels. Diagnosis is primarily based on biochemical evaluation, and surgery is the curative treatment. Imaging techniques like ultrasound and Tc-99m Sestamibi scintigraphy are used for localization, but selective Internal Jugular Venous (SVS) becomes valuable in cases of inconclusive or conflicting results. This study evaluated the diagnostic efficacy of SVS for localizing parathyroid adenomas in cases where non-invasive radiological diagnostic methods yielded inconclusive results or negative findings despite clinical symptoms suggestive of pHPT. *Materials and Methods*: In this retrospective study, a total of 28 patients diagnosed with pHPT underwent SVS at a tertiary center known for receiving referrals from 2017 to 2022. The diagnoses were confirmed through biochemical analysis. The SVS results in 22 patients were compared with non-invasive imaging methods, including ultrasound, scintigraphy, and computed tomography with/without contrast material. SVS was indicated when at least two non-invasive diagnostic procedures failed to clearly localize the parathyroid glands or provided ambiguous results. *Results*: SVS demonstrated higher sensitivity for localizing parathyroid adenomas compared to non-invasive imaging methods, accurately lateralizing the adenoma in 68.18% of cases. Among the SVS findings, 31.8% of patients had negative results, with 9.1% not having clinically proven parathyroid adenoma, while 22.7% had false negative SVS findings but were later confirmed to have adenoma during surgery. Ultrasound correctly identified the location in 45.45% of cases, CT in 27.27%, and scintigraphy in 40.9%. *Conclusions*: SVS is a valuable diagnostic tool for accurately localizing parathyroid adenomas in patients with inconclusive non-invasive imaging results. It aids in targeted surgical interventions, contributing to improved management and treatment outcomes in primary hyperparathyroidism.

## 1. Introduction

Primary hyperparathyroidism (pHPT) emerges as a endocrine disorder, marked by the autonomous and excessive production of parathyroid hormone (PTH). This leads to elevated levels of both PTH and calcium (Ca) due to the impaired regulation of calcium metabolism. It is worth highlighting its status as the third most prevalent endocrine disease, standing out as the primary cause of hypercalcemia observed in outpatient settings [1,2,3].

The diagnosis of pHPT relies primarily meticulous biochemical evaluation. Imaging modalities are deliberately avoided for the purpose of confirming or excluding the diagnosis and determining the necessity for surgery [4]. Presently, there is a discernible inclination toward the widespread adoption of minimally invasive parathyroidectomy (MIP). This preference is attributed to its potential to yield significantly fewer instances of hypocalcemia and recurrent laryngeal nerve injury when compared to the conventional bilateral neck exploration. The advent of MIP has prompted surgeons to increasingly rely on exceedingly precise preoperative localization studies. These studies aim to meticulously pinpoint the position of the functional parathyroid gland and its intricate relationship with surrounding structures [5,6,7,8].

While proper imaging undoubtedly plays a pivotal role in meticulous surgical planning, it is imperative to underscore that it should not be solely relied upon to establish the diagnosis or ascertain the necessity for surgery [9,10,11]. The conventional non-invasive imaging techniques employed for localization encompass ultrasound (US) and Tc-99m Sestamibi scintigraphy (Tc99m MIBI SZ). Simultaneously, magnetic resonance imaging (MRI) and computed tomography (CT) are selectively employed in specific cases. Despite offering commendable detection rates, instances do arise where non-invasive diagnostic methods yield inconclusive or conflicting outcomes [12,13,14].

In scenarios characterized by inconclusive results from non-invasive imaging, Selective venous sampling (SVS) emerges as an invasive yet extraordinarily valuable tool for precise localization. SVS is predominantly reserved for patients grappling with persistent or recurrent pHPT, particularly those with inconclusive results from non-invasive imaging modalities [15]. This intricate procedure entails venous angiography and blood sampling from highly specific locations, including but not limited to the brachiocephalic vein, internal jugular vein, and points where thyroid veins intricately drain into the jugular vein [8,16].

Studies assessing the efficacy of Selective Venous Sampling (SVS) have consistently underscored its superiority over non-invasive methods, revealing higher sensitivity, specificity, and positive likelihood ratios. This comparative advantage becomes particularly evident when confronted with the daunting task of localizing pathological glands that remain elusive using other conventional imaging modalities. In such challenging scenarios, SVS has emerged as a beacon of diagnostic precision, showcasing not only high sensitivity for lateralization but also promising positive predictive value and accuracy rates, as documented in the relevant literature [17,18].

The heightened sensitivity and specificity exhibited by SVS in these instances can be attributed to its ability to directly sample venous blood from the vicinity of the parathyroid glands. This direct sampling provides a more accurate representation of the parathyroid hormone (PTH) levels, offering a dynamic and real-time assessment of gland activity. 

Despite the evident effectiveness of SVS in unraveling complex cases, it is imperative to exercise judiciousness in its application. SVS should be strategically reserved for specific patient populations, particularly those presenting with inconclusive non-invasive diagnostic results. This selective approach ensures that SVS is utilized where its unique capabilities can add significant value, avoiding unnecessary invasive procedures in patients where non-invasive methods prove sufficient. The judicious application of SVS becomes paramount in optimizing patient care, balancing the need for diagnostic precision with the inherent risks and invasiveness associated with the procedure [19].

Therefore, a careful evaluation of each patient’s profile is crucial in determining the appropriateness of SVS, ensuring that it is selectively employed to maximize diagnostic accuracy without subjecting patients to unnecessary procedures.

In such cases, SVS represents a minimally invasive diagnostic option that can be useful in localizing parathyroid gland adenomas. The present study aimed to evaluate the diagnostic efficacy of SVS for lateralizing parathyroid adenomas in cases where non-invasive radiological diagnostic methods yielded inconclusive results or negative findings despite clinical symptoms suggestive of pHPT.

## 2. Materials and Methods

### 2.1. Study Population and Design

This study was conducted in accordance with the ethical standards and received approval from the Institutional Review Board, identified by the reference number 2-PĒK-4/332/2022. All participating patients were thoroughly informed about the invasive procedure scheduled at the Interventional Radiology Department, including potential radiation exposure. Written consent was obtained from each patient before the operation.

The research was carried out at the Interventional Radiology Department of Riga East Clinical University Hospital in Riga, Latvia, a reputable tertiary center known for its expertise in handling referrals. From 2017 to 2022, we enrolled a total of twenty-eight patients who underwent Selective Venous Sampling (SVS) following a clinical diagnosis of primary hyperparathyroidism (pHPT).

Clinical diagnoses were confirmed through rigorous biochemical analysis. To assess the effectiveness of SVS, we compared the results in 22 patients with those obtained through non-invasive imaging methods, including ultrasound, scintigraphy, and computed tomography with/without contrast material. The accuracy of SVS was evaluated by comparing the post-operative material’s localization with SVS data.

SVS was indicated when at least two non-invasive diagnostic procedures failed to clearly localize the parathyroid glands or provided ambiguous results. A positive SVS result was determined by a significant peak in parathyroid hormone (PTH) levels, which had to exceed the peripheral reference value by a factor of 1.5, as obtained during the SVS [20,21]. The sampling site exhibiting the highest peak was subsequently considered the likely location of the hyperactive parathyroid gland.

Exclusion criteria were established to maintain the study’s focus, excluding patients whose primary focus localization could be determined through an ultrasound (USG), CT, and sestamibi scan, as well as those with secondary or tertiary hyperparathyroidism attributed to chronic renal disease.

The SVS result was defined as completely correct if it identified all abnormal glands in the patient. SVS was considered to lateralize the correct side if adenoma was lateralized correctly but no detailed anatomical localization was determined. SVS results were defined as false in following cases: negative finding in patients with parathyroid pathology; false positive result in patients with abnormal gland(s) in a different localization than was determined during SVS; the lateralization of abnormal parathyroid tissue after SVS remains unknown.

A positive noninvasive imaging study demonstrates an enlarged parathyroid gland with typical features (discrete hypoechoic mass with polar vascularization, clear contrast accumulation on scintigraphy, a circumscribed mass on CT that could be distinguished from a lymph node by density). A doubtful result means that there is a suspicious mass that only partially exhibits the above-mentioned features, or the results of different examinations are contradictory. If all these examinations fail to clearly localize the parathyroid adenoma, it served as an indication for SVS.

In this study, the following patient data were investigated: US, scintigraphy, CT findings with/without contrast, SVS results, patho-histological findings of postoperative material, parathyroid hormone (PTH) level in blood before operation/3 days after operation, calcium (Ca) level in blood before operation/1 day after operation/3 days after surgery, blood phosphorus (P) level before surgery, and Ca and P levels in urine before surgery.

The findings of the non-invasive radiological examinations have been compared with the histopathological findings of the surgical material and the results of the interventional radiological examination or SVS.

All patients underwent standard blood tests to confirm hyperparathyroidism. The blood levels of Ca and PTH before and after surgery were compared. Operative treatment was considered successful if the level of both values decreased after surgery, and the histopathological opinion confirmed the presence of parathadenoma. In patients who underwent parathyroidectomy, the obtained operative material was analyzed and sent for histopathological examination to confirm or deny the presence of adenoma.

### 2.2. SVS Technique

Before conducting the SVS, we performed an intraoperative ultrasound (US) of the neck veins and preoperative computed tomography (CT) to identify any potential congenital anatomical variations that might contraindicate SVS or influence the procedural process. Accurate localization and course assessment of veins in the neck are necessary to determine optimal blood sampling points for PTH levels in the internal jugular and brachiocephalic veins. CT, CTA (CT angiography), or MRI scans are typically utilized for this purpose.

The SVS procedure commences with a Seldinger technique, involving a right common femoral vein puncture using either a 5-F or 4-F catheter. Under fluoroscopic guidance, the catheter is advanced to right internal jugular and right brachiocephalic veins venous blood samples are collected from each cervical level with reference to the cervical vertebrae as anatomical markers, allowing for PTH level determination. Approximately 3–5 mL of blood is taken from each neck level for analysis. After completing the right side catheterizations and obtaining venous samples, the same procedure is performed on the left side of the neck.

Finally, a venous sample is taken from the superior vena cava to conclude the SVS manipulation. In total, the number of venous samples collected ranges from 15 to 25, with an average of approximately 17 samples, depending on the type of SVS procedure.

The labeled tube containing the blood sample is immediately cooled. The individual hormone values are then associated with an anatomical diagram based on PTH determination, aiding in distinguishing the location on the left or right side of the neck at various levels.

### 2.3. Statistical Analysis

For the statistical analysis, the Wilcoxon test and quantifiers were employed to compare patients’ data, including US, scintigraphy, CT findings with/without contrast, SVS results, patho-histological findings of postoperative material, parathyroid hormone (PTH) level in blood before operation/3 days after operation, calcium (Ca) level in blood before operation/1 day after operation/3 days after surgery, and blood phosphorus (P) level before surgery. The findings of non-invasive radiological examinations were compared with histopathological findings and the results of interventional radiological examination or SVS.

## 3. Results

In our study, a cohort of 28 female patients, with ages ranging from 37 to 78 years and a mean age of 60 years, underwent a detailed investigation into primary hyperparathyroidism (PHPT). Parathyroidectomy was warranted for 22 patients based on clinical features, data from non-invasive radiological diagnostic methods, and the results obtained from Selective Venous Sampling (SVS). The remaining six patients, not meeting the operative treatment criteria, were subjected to clinical follow-up.

The gender distribution in our study aligns with the common observation of PHPT having a higher incidence in postmenopausal women. This female predominance underscores the relevance of our study in capturing a demographic group that frequently encounters this condition.

**Figure 1:**
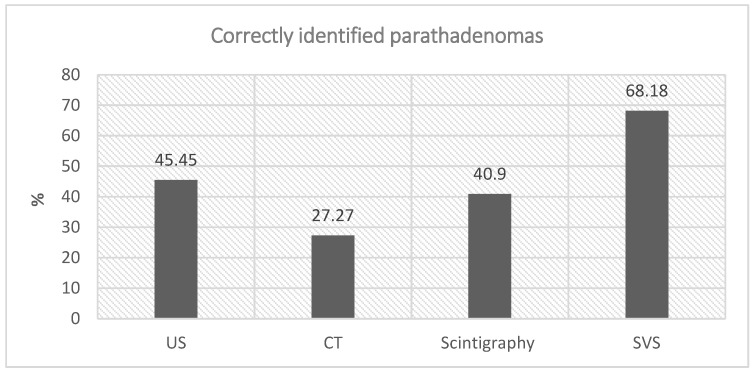
Assessment of Adenoma Localization and Lateralization Methods. Presenting the effectiveness of diverse imaging modalities in both localizing and lateralizing parathyroid adenomas. The data are portrayed as percentages, illustrating the precision of identification for each method and is grounded in a total of 22 cases.

The localization of parathyroid adenomas, a crucial aspect of our investigation, yielded distinct results. Among the non-invasive radiological diagnostic methods employed, ultrasound (US) correctly identified the localization in 10 out of 22 cases (45.45%), computed tomography (CT) in 6 out of 22 cases (27.27%), and scintigraphy in 9 out of 22 cases (40.9%). However, the standout performance was attributed to SVS, which demonstrated superior accuracy in precisely lateralizing the adenoma, correctly identifying it in 15 out of 22 cases (68.18%). This emphasizes SVS as the most accurate preoperative method for determining adenoma lateralization (Figure 1).

**Figure 2:**
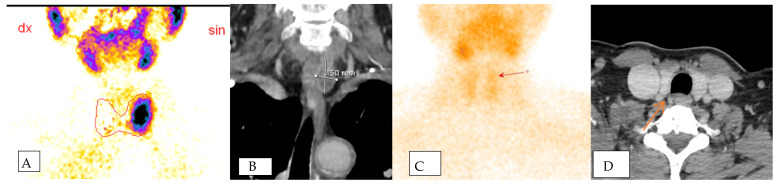
The figure shows positive scintigraphy (**A**) and CT scan (**B**) of the left sided parathadenoma in one patient which localization was confirmed using SVS and postoperative material. Figure (**C**,**D**) shows mismatch between two radiological non-invasive methods. Scintigraphy (**C**) shows suspected left-sided parathadenoma, while CT scan (**D**) shows right-sided parathadenoma. SVS and operative material confirmed left-sided parathadenoma in this patient.

Delving into the SVS results, seven patients showed negative findings. Among these, two did not have clinically proven parathyroid adenomas, while five had false-negative SVS results but were later confirmed to have adenomas intraoperatively. This highlights the dual role of SVS, not only in identifying pathological glands accurately, but also in contributing to the precision of preoperative diagnoses. When SVS correctly lateralized the pathological gland, the PTH gradient was observed to be 1.5–2 times higher than the normal PTH level, providing valuable guidance for localizing the adenoma within the specific anatomical quadrant of the neck (Figure 2).

In terms of blood and urine tests, preoperative calcium (Ca) levels showed a positive statistically significant correlation with preoperative PTH levels (r = 0.403, *p* = 0.033), indicating that higher PTH levels were associated with higher Ca levels. However, there was no statistically significant correlation between preoperative Ca levels and phosphorus (P) levels in the blood or the P/Ca ratio in urine (Figure 1).

After parathyroidectomy, Ca levels on the 1st and 3rd day were statistically significantly lower than preoperative levels (Z = −4.623, *p* ≤ 0.001; Z = −4.272, *p* ≤ 0.001, respectively). The PTH level three days after parathyroidectomy was also statistically significantly lower than the preoperative level (Z = −4.395, *p* ≤ 0.001). These results indicate successful outcomes of parathyroidectomy and clinical recovery of patients (Table 1).

## 4. Discussion

Our results demonstrate that SVS exhibited superior accuracy in preoperative determination of parathyroid adenoma lateralization compared to non-invasive imaging modalities. Specifically, SVS correctly identified the precise lateralization of adenoma in 68.18% of cases, outperforming ultrasound (45.45%), computed tomography (27.27%), and scintigraphy (40.9%).

Although non-invasive imaging studies can often identify lesion locations, they may also yield discordant or inconclusive results after multiple examinations. Previous studies revealed that incorporating SVS alongside non-invasive imaging studies could play a crucial role in enhancing confidence in predictions and improving the accuracy of localization diagnoses [8,22,23].

In a study conducted by Sun et al., the investigation focused on the significance of SVS in the preoperative assessment of patients experiencing persistent (P-PHPT) or recurrent (R-PHPT) primary hyperparathyroidism with nonlocalizing imaging studies. The retrospective analysis, spanning from 2000 to 2014, included 30 patients with P-PHPT or R-PHPT and nonlocalizing noninvasive imaging who underwent SVS. Among them, 12 patients did not proceed to surgical exploration due to negative or non-localizing parathyroid hormone (PTH) gradients (*n* = 8) or chose medical management (*n* = 4). Of the 18 patients who underwent surgical exploration, 94% exhibited a positive PTH gradient, and pathological parathyroid tissue was identified during surgery. The sensitivity and positive predictive value (PPV) of SVS were 93% and 77%, respectively, for all surgical cases, 86% and 60.0% for cervical cases (*n* = 11), and 100% and 100% for mediastinal cases (*n* = 7). The study concluded that SVS proves valuable in cases of P-PHPT or R-PHPT with nonlocalizing imaging studies, providing a high sensitivity and PPV, ultimately contributing to the successful surgical cure in the majority of patients (89%) [22].

Between January 1990 and December 2001, Julija JJ and colleagues conducted a study involving 235 patients with persistent or recurrent hyperparathyroidism. Out of these, 27% (64 patients) were identified as candidates for SVS in conjunction with noninvasive localization studies. Upon retrospectively evaluating SVS accuracy for parathyroid hormone (PTH), the study found that successful surgical treatment was achieved in 86% of these patients. SVS for PTH exhibited true-positive results in 75% of cases, proving its value for surgeons. However, it was deemed not useful in 17% of cases, with 12% being completely false-positive and 5% indeterminate. Notably, SVS successfully identified one abnormal gland in 2% of patients, but failed to locate another abnormal gland. In 6% of cases, the location of abnormal parathyroid tissue remained unknown, resulting in persistent hyperparathyroidism in these patients. In conclusion, the study underscores the clinical utility of SVS in instances of persistent or recurrent hyperparathyroidism, particularly when noninvasive localization studies fall short of clearly identifying the abnormal parathyroid glands [23].

Our results are consistent with these prior investigations that have highlighted the diagnostic superiority of SVS in challenging cases where standard imaging techniques may fail to provide adequate localization information.

Based on Yamada et al., in cases of recurrent or persistent hyperparathyroidism necessitating a second operation, SVS has shown a high sensitivity value, ranging from 75% to 94.7%, in detecting the specific area of ectopic parathyroid tissue [24].

While our positive results with SVS compared to non-invasive imaging modalities are noteworthy, it is important to acknowledge that there are studies contradicting the efficacy of internal jugular vein sampling. For example, Alvarado et al. demonstrated that bilateral internal jugular venous sampling did not markedly improve the accuracy of localizing parathyroid adenomas in primary hyperparathyroidism (PHPT), especially in instances involving negative Sestamibi scans [25].

The majority of cases of primary hyperparathyroidism (PHPT), around 85%, result from single gland disease. As a result, focused approaches are often adequate for treatment instead of resorting to a four-gland exploration. Therefore, the key factor lies in accurately localizing the lesion [26]. Clearly, there are new techniques for localizing parathyroid adenoma. However, in clinical practice, these innovative methods like 4D-CT or PET with 11C Methionine or Choline are not yet widely adopted. In cases where conventional methods fail, SVS remains a viable option for localizing parathyroid adenoma in the neck [27,28].

According to a previous meta-analysis, SVS exhibited superior sensitivity compared to non-invasive imaging modalities in re-operative parathyroid surgery.

Eloy JA et al. in a retrospective analysis of fourteen patients with nonlocalizing parathyroid adenomas post-ultrasonography, Sestamibi scanning, and MRI, eight patients underwent SVS before surgery, while six patients opted for a traditional four-gland neck exploration without preoperative SVS. The study reveals that all patients underwent successful, uncomplicated surgical parathyroidectomy. SVS demonstrated a high accuracy rate in preoperatively localizing the adenoma in 87% of patients, enabling a minimally invasive approach. The mean operative time for the SVS group was notably shorter at 33 min compared to 67 min in the non-SVS group. Importantly, no procedural complications were reported in either group [17].

However, the invasive nature of SVS limits its feasibility for routine use. Nevertheless, SVS can be considered as a valuable imaging test in primary cases when conventional imaging results are negative, enabling a focused approach and reducing the risk of persistency. Additionally, there have been reports suggesting that combining parathyroid SVS with 4D-CT enhances the sensitivity and accuracy in identifying parathyroid adenoma in patients with recurrent or persistent primary hyperparathyroidism (PHPT) and negative findings on scintigraphy and ultrasound scans. These reports found that when comparing 4D-CT alone with 4D-CT plus SVS, the inclusion of SVS resulted in a significant increase in both sensitivity and accuracy [29,30].

While SVS is associated with drawbacks such as being time-consuming, expensive, and carrying a risk of infection, it has demonstrated higher sensitivity and accuracy in localizing the lesion than non-invasive methods. It has even been found to increase the success rate of second surgeries [18,30,31].

Despite the time-consuming and technically demanding nature of catheterizing multiple thyroid veins, numerous studies have highlighted the significant benefits of selectively collecting samples from the thyroid vein whenever possible. The thyroid veins have shown the most substantial iPTH gradient, and conducting SVS on these veins provides detailed spatial resolution, potentially resulting in higher sensitivity when selectively sampled [8,20,32]. However, the objective of our study was to precisely demonstrate the outcomes of selective internal jugular vein sampling, which, in contrast to superselective thyroid sampling, is considerably quicker and involves lower radiation and contrast doses.

SVS proved highly informative when it correctly lateralized pathological glands, with a PTH gradient consistently higher than the normal PTH level, thereby allowing the determination of potential anatomical quadrants in the neck where the adenoma might be located.

It is noteworthy that while SVS exhibited favorable accuracy in correctly lateralizing pathological glands, our study also identified certain limitations in its diagnostic performance. Specifically, seven patients in our cohort had negative SVS findings, with five of them showing false negative results despite being confirmed to have adenomas during intraoperative examination. Similar occurrences of false negative SVS findings have been reported in other studies [8,17,27], highlighting the importance of meticulous procedural technique and careful interpretation of results. Such discrepancies may be attributed to technical factors during the SVS procedure or variations in the pathological gland’s PTH gradient that affected detection.

In assessing the blood and urine tests, we observed a significant positive correlation between preoperative Ca levels and PTH levels, indicating that higher PTH levels were associated with elevated Ca levels. This finding supports the established physiological relationship between PTH and Ca regulation in PHPT. However, we did not observe a significant correlation between preoperative Ca levels and blood P levels or the P/Ca ratio in urine. This may be influenced by other factors affecting phosphorus homeostasis in PHPT patients, warranting further investigation.

Furthermore, the postoperative analysis revealed a statistically significant decrease in Ca levels on the 1st and 3rd day after parathyroidectomy, along with a substantial reduction in PTH levels on the 3rd day. These results indicate a successful surgical outcome, with effective resolution of hypercalcemia and normalization of PTH levels. The observed clinical recovery of patients reinforces the curative potential of parathyroidectomy as the definitive treatment for PHPT.

The primary drawbacks associated with SVS encompass its high cost, necessitating experienced personnel and the involvement of radiation exposure. Additionally, SVS may give rise to certain complications, including groin hematomas, vascular injury, and venous thrombosis [8,24,33,34]. However, it is noteworthy that none of our patients encountered any complications related to SVS during the course of this study.

Our study has several limitation factors: first. the retrospective nature of the study and the lack of randomization in assigning patients to different diagnostic procedures (SVS vs. non-invasive imaging methods) introduce the possibility of confounding factors. Differences in patient characteristics, disease severity, or other variables between the groups could influence the study’s outcomes. Second, despite being a minimally invasive diagnostic procedure, SVS is not without its limitations. The technique relies on sampling venous blood from specific locations in the neck to determine parathyroid hormone levels. However, the spatial distribution of parathyroid hormone might not be uniform, leading to potential sampling errors and inaccurate localization of hyperactive parathyroid glands. Moreover, anatomical variations or technical difficulties during the SVS procedure could further impact its accuracy. Finally, the limited scope of non-invasive imaging methods, the study compares SVS results with those of non-invasive imaging methods, including ultrasound, scintigraphy, and computed tomography. However, there are other emerging imaging modalities, such as 4D-CT, which have shown promise in parathyroid gland localization. Excluding these newer techniques from the study may limit the assessment of the full spectrum of available diagnostic options for hyperparathyroidism.

The main limitation of our study is the lack of PET Choline data and intraoperative parathyroid hormone (iPTH) assay information. This limitation arises primarily from resource constraints, which prevented us from accessing PET Choline imaging and intraoperative PTH assays, thereby impeding our ability to obtain comprehensive data on these specific methodologies.

## 5. Conclusions

Our study showed the diagnostic value of SVS in localizing parathyroid adenomas, particularly when non-invasive imaging methods yield inconclusive or negative results. SVS has shown promising accuracy in lateralizing adenomas, supporting its role as a valuable adjunct to preoperative localization strategies. However, careful consideration of technical factors and patient characteristics is crucial in achieving optimal diagnostic outcomes. Further research is warranted to refine and optimize the SVS technique, ultimately contributing to enhanced surgical precision and patient care in the management of pHPT.

## Figures and Tables

**Table 1:** Pre and Postoperative Calcium (Ca) and PTH Levels and Changes.

Parameters	Mean ± SD (*n* = 22), n	*p*
Preoperative Ca level (mmol/L)	2.86 ± 0.05	<0.001
1-day postoperative Ca level (mmol/L)	2.57 ± 0.04	0.334
3 days postoperative Ca level (mmol/L)	2.42 ± 0.05	0.580
Decrease in Ca level after 1-day postoperative	0.31 ± 0.07	<0.001
Decrease in Ca level after 3 days postoperative	0.52 ± 0.09	<0.001
PTH level preoperative (pg/mL)	288.31 ± 70.15	<0.001
3 days postoperative PTH level (pg/mL)	72.47 ± 16.67	<0.001
Decrease in PTH level after 3 days postoperative	276.54 ± 90.90	<0.001

## Data Availability

Data available upon request from corresponding author.
